# Postpandemic Trends in Pediatric Respiratory Virus Hospitalizations: A Multicenter Study, 2019–2025

**DOI:** 10.1111/irv.70296

**Published:** 2026-09-28

**Authors:** Federica Attaianese, Silvia Campani, Sandra Trapani, Ilaria Alberti, Maurizio Aricò, Marina Attanasi, Giulia Bertolucci, Silvia Bressan, Désirée Caselli, Veronica Casotto, Salvatore Cazzato, Francesco Chiarelli, Enrico Felici, Anna Frusciante, Maria Antonia Galeone, Silvia Garazzino, Antonietta Giannattasio, Eloisa Gitto, Antonio Guerriero, Fiorentina Guida, Giovanna Iudica, Marcello Lanari, Claudia Mandato, Alice Manzini, Gaia Martelli, Gregorio Paolo Milani, Maria Moriondo, Lucia Dora Notarangelo, Felice Nunziata, Roberta Pellegrino, Diego Peroni, Emanuela Piccotti, Eduardo Ponticiello, Silvia Ricci, Immacolata Rulli, Fabio Savoia, Erika Silvestro, Antonella Sisto, Federica Soro, Stefania Tonetto, Alessandro Zago, Carlo Dani, Chiara Azzari, Giuseppe Indolfi

**Affiliations:** ^1^ Meyer Children's Hospital IRCCS Pediatric Unit Florence Italy; ^2^ Department of Health Science University of Florence Florence Italy; ^3^ IRCCS Ca' Granda Ospedale Maggiore Policlinico Milan Italy; ^4^ Pediatric Unit S. Spirito Hospital, Azienda Sanitaria Locale di Pescara Pescara Italy; ^5^ Interdisciplinary Department of Medicine Aldo Moro University of Bari Bari Italy; ^6^ Department of Pediatrics University of Chieti Chieti Italy; ^7^ Pediatric Unit, Pneumonology and Allergology Section Pisa University Hospital Pisa Italy; ^8^ Pediatric Emergency Department University of Padova Padova Italy; ^9^ Department of Woman's and Child's Health University of Padova Padova Italy; ^10^ Pediatric Infectious Diseases Unit AOUC Policlinico di Bari, Children Hospital Giovanni XXIII Bari Italy; ^11^ Pediatric Unit, Department of Mother and Child Health Salesi Children's Hospital Ancona Italy; ^12^ Pediatric and Pediatric Emergency Unit, Children's Hospital AO SS Antonio e Biagio e C. Arrigo Alessandria Italy; ^13^ Medical Directorate Ruggi d'Aragona University Hospital Salerno Italy; ^14^ Pediatric Unit Sant'Anna e San Sebastiano Hospital Caserta Italy; ^15^ Infectious Diseases Unit, Regina Margherita Children's Hospital University of Turin Turin Italy; ^16^ Pediatric Emergency Unit Santobono‐Pausilipon Children's Hospital Naples Italy; ^17^ Neonatal and Pediatric Intensive Care Unit, G. Martino Hospital, Department of Clinical and Experimental Medicine University of Messina Messina Italy; ^18^ Department of Medicine, Surgery and Dentistry “Scuola Medica Salernitana”, Pediatrics Section, University of Salerno Salerno Baronissi Italy; ^19^ Pediatric Unit IRCCS Azienda Ospedaliero‐Universitaria di Bologna Bologna Italy; ^20^ Department of Medical and Surgical Sciences, Alma Mater Studiorum University of Bologna Bologna Italy; ^21^ Department of Health Sciences (DISSAL), University of Genoa Genoa Italy; ^22^ Postgraduate School in Hygiene and Preventive Medicine University of Brescia Brescia Italy; ^23^ Department of Clinical Science and Community Health University of Milan Milan Italy; ^24^ Pediatric Immunology Unit Meyer Children's Hospital IRCCS Florence Italy; ^25^ Medical Direction, Children's Hospital ASST Spedali Civili Brescia Italy; ^26^ Pediatric Unit S. Giuseppe Moscati Hospital Avellino Italy; ^27^ Pediatric Emergency Unit IRCCS Giannina Gaslini Institute Genoa Italy; ^28^ Childhood Cancer Registry of Campania Santobono‐Pausilipon Children's Hospital Naples Italy; ^29^ University of Trieste Trieste Italy; ^30^ Neonatal Intensive Care Unit, Division of Neonatology Careggi University Hospital Florence Italy; ^31^ Department of Neurosciences, Psychology, Drug Research and Child Health University of Florence Florence Italy

**Keywords:** epidemiology, influenza, pediatric hospitalization, respiratory tract infections, RSV, seasonality

## Abstract

**Background:**

The COVID‐19 pandemic profoundly altered the circulation of respiratory viruses in children, changing the epidemiology of acute lower respiratory tract infections. In the postpandemic period, respiratory pathogens showed marked temporal variability. The 2024–2025 season was the first respiratory syncytial virus (RSV) season in Italy during which RSV immunoprophylaxis with nirsevimab was implemented, allowing evaluation of recent trends in pediatric hospitalizations.

**Methods:**

We conducted a retrospective multicenter study across 16 pediatric hospitals in Italy. Hospitalizations of children younger than 18 years with RSV, influenza, adenovirus, parainfluenza virus, rhinovirus infection, or bronchiolitis of unspecified viral etiology were identified from January 2019 to March 2025 using ICD‐9‐CM diagnostic codes. Annual and monthly hospitalization rates per 100,000 children were calculated using national demographic data. Temporal trends were assessed across six consecutive seasons.

**Results:**

Overall, 23,981 hospitalizations were recorded. RSV was the most frequent pathogen (42.5%), followed by influenza (18.6%), adenovirus (9.9%), and rhinovirus (7.3%). Hospitalizations declined sharply in 2020 and subsequently increased, peaking in 2024 (*n* = 4903; +30.8% vs. 2022). During the 2024–2025 season, total admissions decreased by 17.2% compared with 2023–2024 (3390 vs. 4095), mainly due to a marked reduction in RSV‐related hospitalizations (−41.6%, 1230 vs. 2107), with modest increases in influenza‐, adenovirus‐, and rhinovirus‐associated admissions.

**Conclusions:**

This nationwide analysis describes postpandemic trends in pediatric respiratory virus hospitalizations in Italy, highlighting changing pathogen‐specific patterns and the need for continued viral surveillance.

**Abbreviations**
ALRTI: acute lower respiratory tract infection
AOPI: Associazione Ospedali Pediatrici Italiani
CI: confidence interval
ICD‐9‐CM: International Classification of Diseases, 9th Revision, Clinical Modification
ISTAT: Italian National Institute of Statistics
NPI: nonpharmaceutical intervention
RSV: respiratory syncytial virus

## 1 Introduction

Acute lower respiratory tract infections (ALRTIs) remain a major cause of pediatric morbidity and mortality worldwide, and place a substantial burden on healthcare systems [1, 2]. In 2021, an estimated 37.8 million ALRTI episodes occurred globally in children under 5 years, corresponding to an incidence of 5750 episodes per 100,000 children [2]. Viruses are the predominant etiologic agents of ALRTIs. Respiratory syncytial virus (RSV) is the leading pathogen, responsible for more than 3.6 million hospitalizations globally among children younger than 5 years in 2019, including 1.4 million in infants aged 0–6 months [3, 4, 5]. Influenza, adenovirus, rhinovirus, and parainfluenza viruses also contribute significantly to the global burden of pediatric acute respiratory disease [2, 4, 6, 7, 8]. Since early 2020, the COVID‐19 pandemic and the widespread implementation of nonpharmaceutical interventions (NPIs) profoundly altered the epidemiology and seasonal dynamics of respiratory viruses [3, 9]. Several studies have reported unprecedented declines in RSV and influenza activity, followed by atypical resurgences and shifts in seasonal timing once NPIs were lifted [9]. Previous data from the “Associazione Ospedali Pediatrici Italiani” (AOPI) network, a collaboration of major Italian pediatric hospitals, similarly documented substantial disruptions in viral seasonality through the 2019–2023 period [4]. More recently, nirsevimab, a long‐acting monoclonal antibody for RSV prevention, has been introduced in several countries. Early real‐world data suggest reductions in RSV‐related hospitalizations among infants [10, 11, 12, 13, 14]. In Italy, nirsevimab was approved in January 2023 and implemented during the 2024–2025 season. However, its broader epidemiologic implications, particularly regarding non‐RSV respiratory viruses, remain uncertain. The reduction in RSV circulation may theoretically influence the epidemiology of other respiratory viruses through ecological mechanisms, including viral interference, changes in the pool of susceptible hosts, and healthcare‐system dynamics [4, 9, 15]. However, evidence supporting these effects remains limited. This study expands upon previous national analyses by providing one of the first multicenter assessments of postpandemic pediatric respiratory virus dynamics across six consecutive seasons (2019–2025). By examining temporal trends across major viral pathogens, we aim to characterize evolving seasonal patterns and describe how the landscape of pediatric respiratory infections is changing in the postpandemic era.

## 2 Methods

### 2.1 Study Design and Population

We conducted a multicenter retrospective observational study across 16 pediatric wards, each located in a separate hospital, distributed across 12 Italian regions, and covering all major geographic areas of the country. Participating centers included major pediatric referral hospitals and university‐affiliated pediatric units, selected based on availability of ICD‐9‐CM‐coded electronic discharge records and existing collaborative networks. As centers were recruited through convenience sampling within established research networks rather than systematic nationwide recruitment, first‐level pediatric units are not represented, which may limit generalizability to smaller hospital settings.

All hospitalizations of children and adolescents (< 18 years) related to RSV, influenza, parainfluenza, rhinovirus, adenovirus infection, or bronchiolitis of unspecified viral etiology, occurring between January 2019 and March 2025, were identified through the International Classification of Diseases, 9th Revision, Clinical Modification (ICD‐9‐CM) diagnostic codes. These included the following: acute bronchiolitis due to RSV (ICD‐9‐CM 466.11), RSV infection (ICD‐9‐CM 079.6), and RSV pneumonia (ICD‐9‐CM 480.1); influenza with pneumonia (ICD‐9‐CM 487.0), influenza with other respiratory manifestations (ICD‐9‐CM 487.1), and influenza with other manifestations (ICD‐9‐CM 487.8); adenovirus infection in diseases classified elsewhere (ICD‐9‐CM 079.0) and adenovirus pneumonia (ICD‐9‐CM 480.0); rhinovirus infections in diseases classified elsewhere and of unspecified site (ICD‐9‐CM 079.3); parainfluenza virus pneumonia (ICD‐9‐CM 480.2); and acute bronchiolitis due to other infectious agents (ICD‐9‐CM 466.19), stated in tables and figures as “Others”. The study is reported in accordance with the STROBE guidelines (Strengthening the Reporting of Observational Studies in Epidemiology). During the preparation of this work, the authors used ChatGPT‐4 (OpenAI, March 2025) to improve language clarity and grammar. All content was reviewed and edited by the authors, who take full responsibility for the final version.

### 2.2 Statistical Analysis

Descriptive analyses were performed to evaluate overall and pathogen‐specific hospitalizations by year and month. Frequencies, proportions, and hospitalization rates per 100,000 children were calculated. Rates were standardized using the population aged < 18 years residing in the regions of the participating centers, based on the Italian National Institute of Statistics (Istituto Nazionale di Statistica, ISTAT) demographic data for 2022–2024, with annual adjustments. Hospitalization rates were calculated using regional pediatric population denominators to allow temporal comparisons across years. Because some participating hospitals are tertiary referral centers and may receive patients from outside their immediate geographical area, these rates should be interpreted as standardized hospitalization rates rather than population‐based incidence estimates. Temporal trends were explored through monthly counts to assess seasonal patterns and interannual variability. Year‐to‐year changes were expressed as relative percentage increases or decreases compared with the previous year, whereas percentages reported in the text for individual pathogens represent their proportion of total hospitalizations unless otherwise specified. Ninety‐five percent confidence intervals (95% CIs) for hospitalization rates were calculated assuming a Poisson distribution of monthly event counts, using exact methods.

## 3 Results

### 3.1 General and Relative Hospitalization Trends

Between January 2019 and March 2025, the 16 participating pediatric centers documented a total of 23,981 hospitalizations for acute viral respiratory tract infections. RSV was the leading pathogen, accounting for 42.5% of all hospitalizations (*n* = 10,192), followed by influenza (18.6%, *n* = 4463), adenovirus (9.9%, *n* = 2373), rhinovirus (7.3%, *n* = 1744), and parainfluenza virus (1.4%, *n* = 324) (Figure 1). Acute bronchiolitis attributable to other infectious agents represented 20.4% of the total (*n* = 4484) (Figure 1). A sharp decline in hospitalizations was observed in 2020 (*n* = 2617), coinciding with the onset of the COVID‐19 pandemic, compared with 2019 (*n* = 3600). From 2020 onward, hospitalizations rose steadily, peaking in 2023 (+945 cases; +28.0% vs. 2022) and exceeding the numbers recorded in 2019. (Figure 1). The peak was reached in 2024 (*n* = 4903), exceeding 2019 levels by 1303 cases and those recorded in 2020 by 2286 cases (Figure 1). Hospitalization rates per 100,000 children under 18 years rose from 58.2 in 2022 to 87.4 in 2024, with the most pronounced increase between 2022 and 2023 (+30.8%) (Table 1 and Figure 2).

**FIGURE 1:**
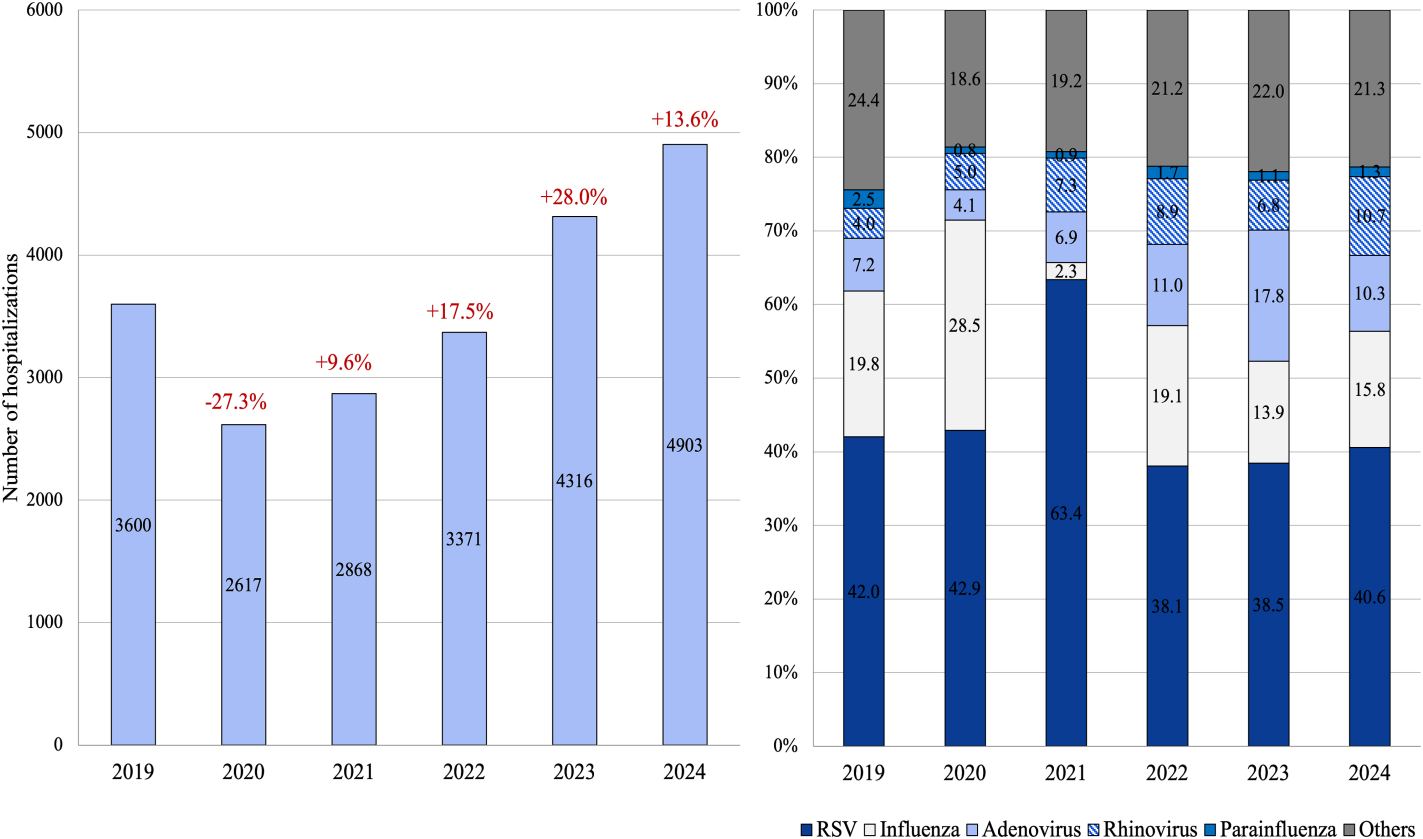
Annual hospitalizations for ARIs (left panel) and proportion of admissions attributable to each virus between 2019 and 2025 (right panel). RSV and influenza accounted for the largest share, with year‐to‐year fluctuations observed across all pathogens. ARIs: acute respiratory infections. RSV: respiratory syncytial virus.

**TABLE 1:** Annual and seasonal pediatric hospitalizations for respiratory viruses, hospitalization rates per 100,000 children, and year‐on‐year variation.

	Year/autumn–winter season^a^	RSV	Influenza	Adenovirus	Rhinovirus	Parainfluenza	Others	Total
Number of hospitalization (%)/year	2019	1513 (42.0)	714 (19.8)	258 (7.2)	145 (4.0)	91 (2.5)	879 (24.4)	3600 (100)
	2020	1123 (42.9)	747 (28.5)	108 (4.1)	130 (5.0)	22 (0.8)	487 (18.6)	2617 (100)
	2021	1818 (63.3)	66 (2.3)	198 (6.9)	209 (7.3)	26 (0.9)	551 (19.2)	2868 (100)
	2022	1283 (38.0)	644 (19.1)	372 (11.0)	300 (8.9)	57 (1.7)	715 (21.2)	3371 (100)
	2023	1660 (38.5)	598 (13.9)	768 (17.8)	293 (6.8)	49 (1.1)	948 (22.0)	4316 (100)
	2024	1989 (40.6)	775 (15.8)	506 (10.3)	524 (10.7)	65 (1.3)	1044 (21.3)	4903 (100)
Number of hospitalization (%)/Autumn–winter season^a^	2023–2024	2107 (51.4)	768 (18.7)	259 (6.3)	203 (5.0)	31 (0.8)	727 (17.7)	4095 (100)
	2024–2025	1230 (36.3)	1021 (30.1)	317 (9.3)	355 (10.5)	30 (0.9)	437 (12.9)	3390 (100)
Hospitalization rate/100.000 (95% CI)	2022	22.1 (20.9–23.4)	11.1 (10.3–12.0)	6.4 (5.8–7.1)	5.2 (4.6–5.8)	1.0 (0.75–1.30)	12.3 (11.4–13.2)	58.2 (56.2–60.3)
	2023	29.1 (27.7–30.5)	10.5 (9.7–11.4)	13.5 (12.6–14.5)	5.1 (4.5–5.7)	0.9 (0.67–1.17)	16.6 (15.6–17.7)	75.7 (73.4–78.0)
	2024	35.4 (33.9–36.9)	13.8 (12.8–14.8)	9.0 (8.2–9.8)	9.3 (8.6–10.2)	1.2 (0.92–1.52)	18.6 (17.5–19.7)	87.4 (85.0–89.8)
Year‐on‐year percentage difference (%)	2022–2023	31.4	−5.7	109.7	−0.8	−12.7	34.7	30.0
	2023–2024	21.8	31.7	−33.0	81.8	34.8	11.9	15.4
	2022–2024	60.0	24.3	40.4	80.3	17.8	50.8	50.2

Abbreviations: CI, confidence interval; RSV, respiratory syncytial virus.

^a^ Autumn–winter season: October 1 to March 31.

**FIGURE 2:**
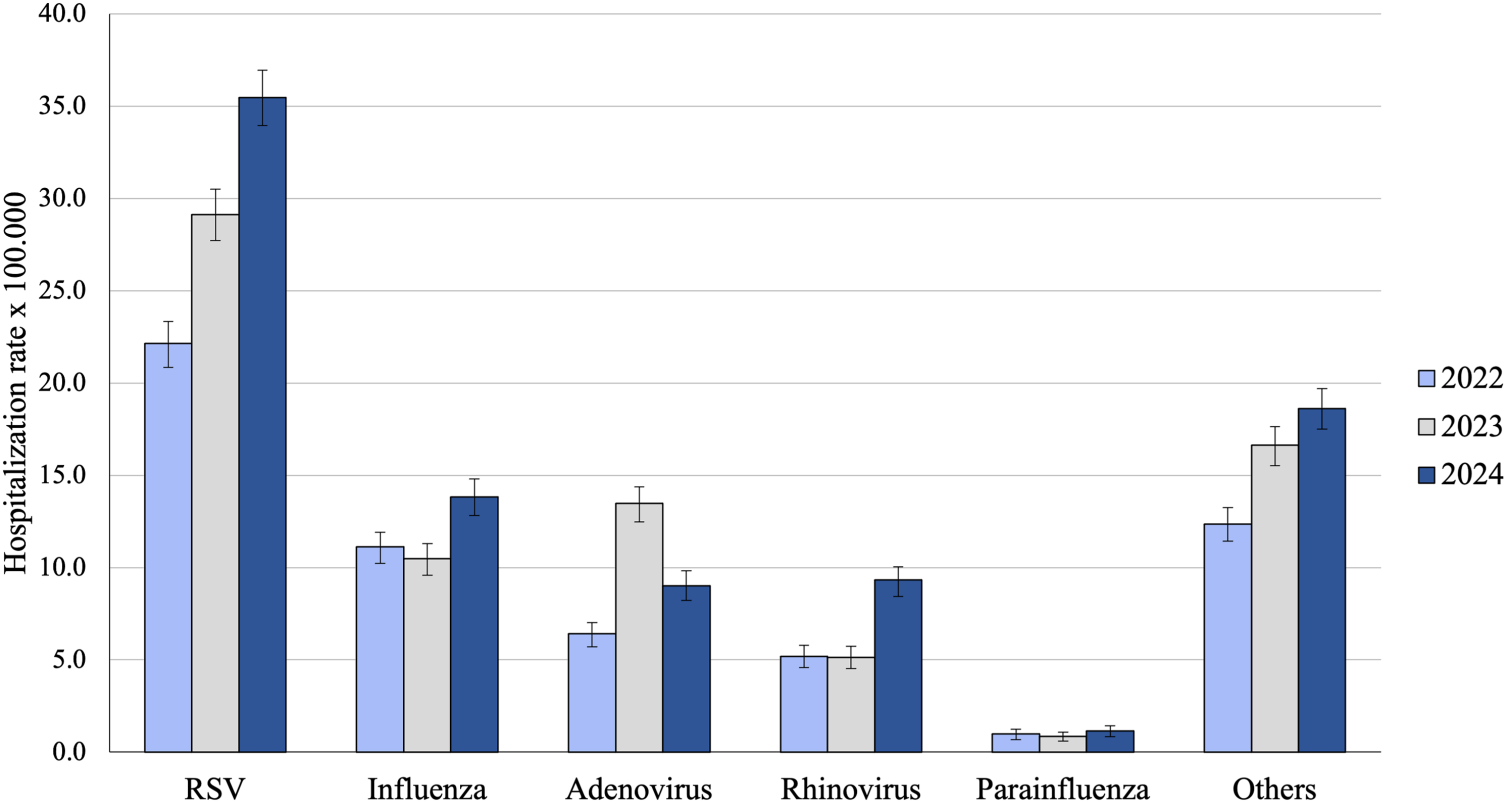
Hospitalization rates per 100,000 population for major respiratory viruses from 2022 to 2024. Error bars indicate 95% confidence intervals calculated using Poisson exact methods. RSV: respiratory syncytial virus.

### 3.2 Pathogen‐Specific Hospitalization Trends

#### 3.2.1 RSV

RSV remained the leading cause of pediatric hospitalization throughout the study period. Hospitalization rates increased from 22.2 per 100,000 in 2022 to 35.5 per 100,000 in 2024, corresponding to a 59.9% increase in hospitalization rate over the 2022–2024 period. Following the January–February 2020 peak, admissions declined markedly during the first pandemic year and remained low until autumn 2021, when an atypical early resurgence was observed. Subsequent epidemic peaks occurred during the winter months of 2022–2024, although seasonal timing progressively shifted toward later months. During the 2024–2025 season, RSV hospitalizations decreased substantially compared with the previous season (1230 vs. 2107 admissions; −41.6%), with a markedly lower epidemic peak (Figure 3).

**FIGURE 3:**
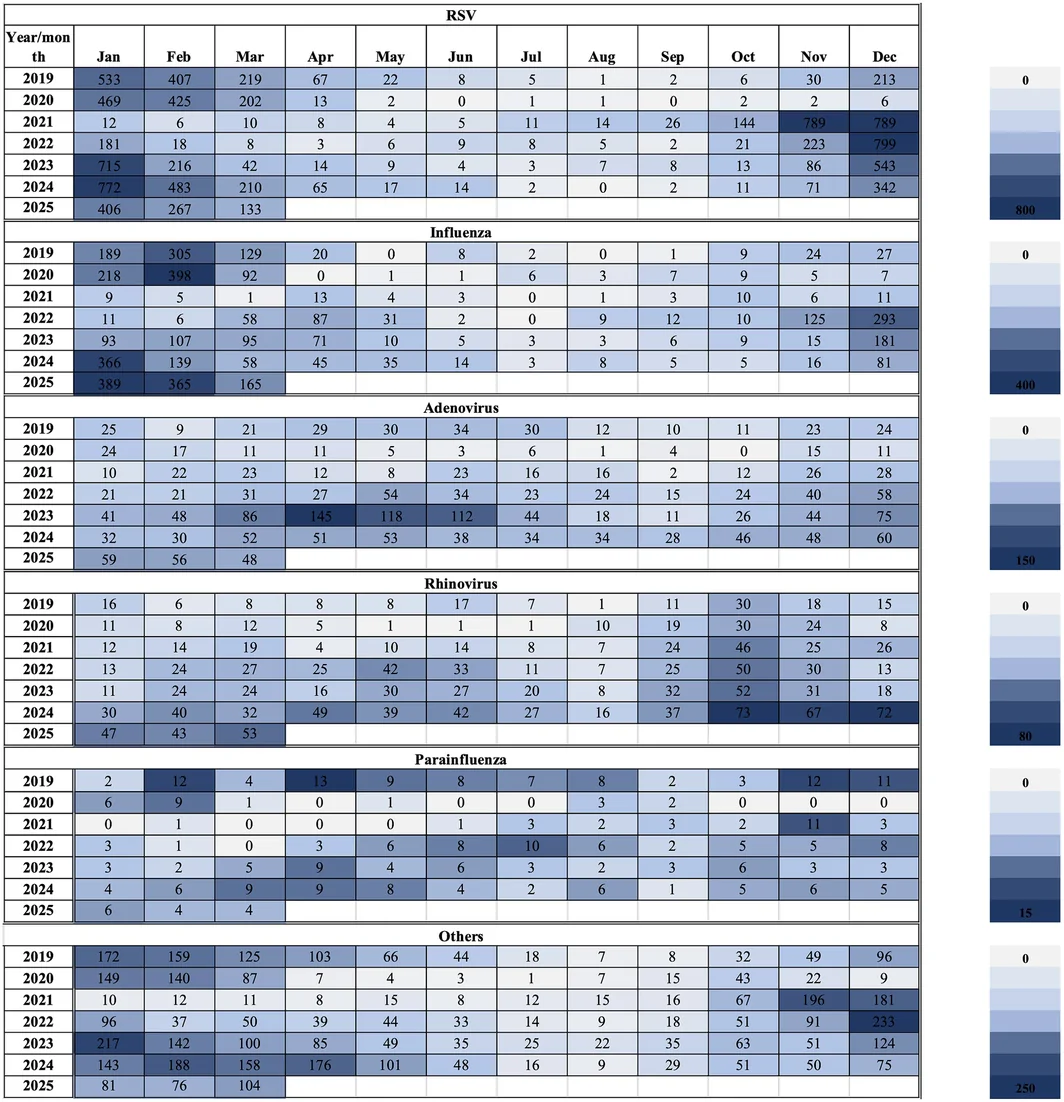
Monthly hospitalizations for respiratory syncytial virus (RSV), influenza, adenovirus, rhinovirus, parainfluenza, and other respiratory viruses from January 1, 2019 through March 31, 2025. Each panel is displayed as a heatmap, with darker shading indicating higher hospitalization counts. Because hospitalization volumes differ substantially across pathogens, each heatmap uses an independent color scale, shown to the right of each panel.

#### 3.2.2 Influenza Virus

Influenza was the second most frequent cause of hospitalization. After an almost complete disappearance during 2020–2021, activity gradually returned, with hospitalization rates increasing from 11.1 to 13.8 per 100,000 children between 2023 and 2024 (+31.7%). Seasonal peaks generally occurred during winter months, although timing varied across years, with earlier circulation in 2022 and a later peak during the 2024–2025 season. Compared with the previous season, influenza hospitalizations increased by 33%, approaching prepandemic levels (Figure 3).

#### 3.2.3 Adenovirus

Adenovirus showed substantial interannual variability and lacked a consistent seasonal pattern. Following the marked decline observed during the pandemic, hospitalization rates increased from 6.4 per 100,000 children in 2022 to a peak of 13.5 per 100,000 in 2023 (+109.8%). Unlike RSV and influenza, adenovirus activity was frequently characterized by spring and summer increases, and hospitalization levels remained above prepandemic values through 2025 (Figure 3).

#### 3.2.4 Rhinovirus

Rhinovirus‐related hospitalizations showed a progressive increase throughout the study period. In contrast to RSV and influenza, rhinovirus circulation appeared less disrupted by the pandemic and maintained its characteristic autumn predominance. Hospitalization rates nearly doubled between 2023 and 2024 (5.1 vs. 9.3 per 100,000; +81.8%), with sustained activity extending beyond the usual seasonal peak during 2024–2025 (Figure 3).

#### 3.2.5 Parainfluenza Virus

Parainfluenza remained the least frequently detected pathogen throughout the study period. Although relative fluctuations were observed across years, absolute hospitalization numbers remained low. After the marked decline observed during the pandemic, circulation resumed with irregular seasonal patterns characterized by occasional spring and summer increases rather than consistent winter peaks (Figure 3).

#### 3.2.6 Acute Bronchiolitis due to Other Infectious Agents

Hospitalizations for bronchiolitis attributed to other infectious agents represented approximately one‐fifth of all admissions throughout the study period. Following a sharp reduction during the pandemic, activity resumed from late 2021 onwards, with seasonal peaks generally occurring during autumn and winter. Admissions remained substantial during the study period, although less pronounced during the 2024–2025 season (Figure 3).

### 3.3 Autumn–Winter Season 2024–2025

The 2024–2025 autumn–winter season (from October 2024 to March 2025) showed marked differences compared with previous years. Total hospitalizations declined by 17.2% compared with 2023–2024. Despite remaining the predominant cause of admission, RSV accounted for 36.3% of all hospitalizations during the 2024–2025 season (*n* = 1230), compared with 51.5% during the 2023–2024 season (*n* = 2107). Influenza accounted for 1021 admissions (30.1%), followed by rhinovirus (*n* = 355, 10.5%), adenovirus (*n* = 317, 9.4%), parainfluenza (*n* = 30, 0.9%), and other unspecified viruses (*n* = 437, 12.9%). A monthly analysis highlighted a relative reduction of 41.6% in RSV admissions compared with the previous season, with the 2025 peak almost halved compared to 2024 (*n* = 406, −47.4%). By contrast, influenza admissions rose by 33%, with a higher seasonal peak (*n* = 389, +6.3%) and sustained increases in February (*n* = 365, +162%) and March (*n* = 165, +189%). Admissions due to adenovirus (+22.4%) and rhinovirus (+74.9%) also increased, whereas admissions due to parainfluenza and other unspecified infectious agents declined (Figure 4). Age‐specific analyses were not available; therefore, observed changes reflect overall pediatric hospitalization patterns and cannot be directly attributed to effects within the infant population targeted by nirsevimab immunization.

**FIGURE 4:**
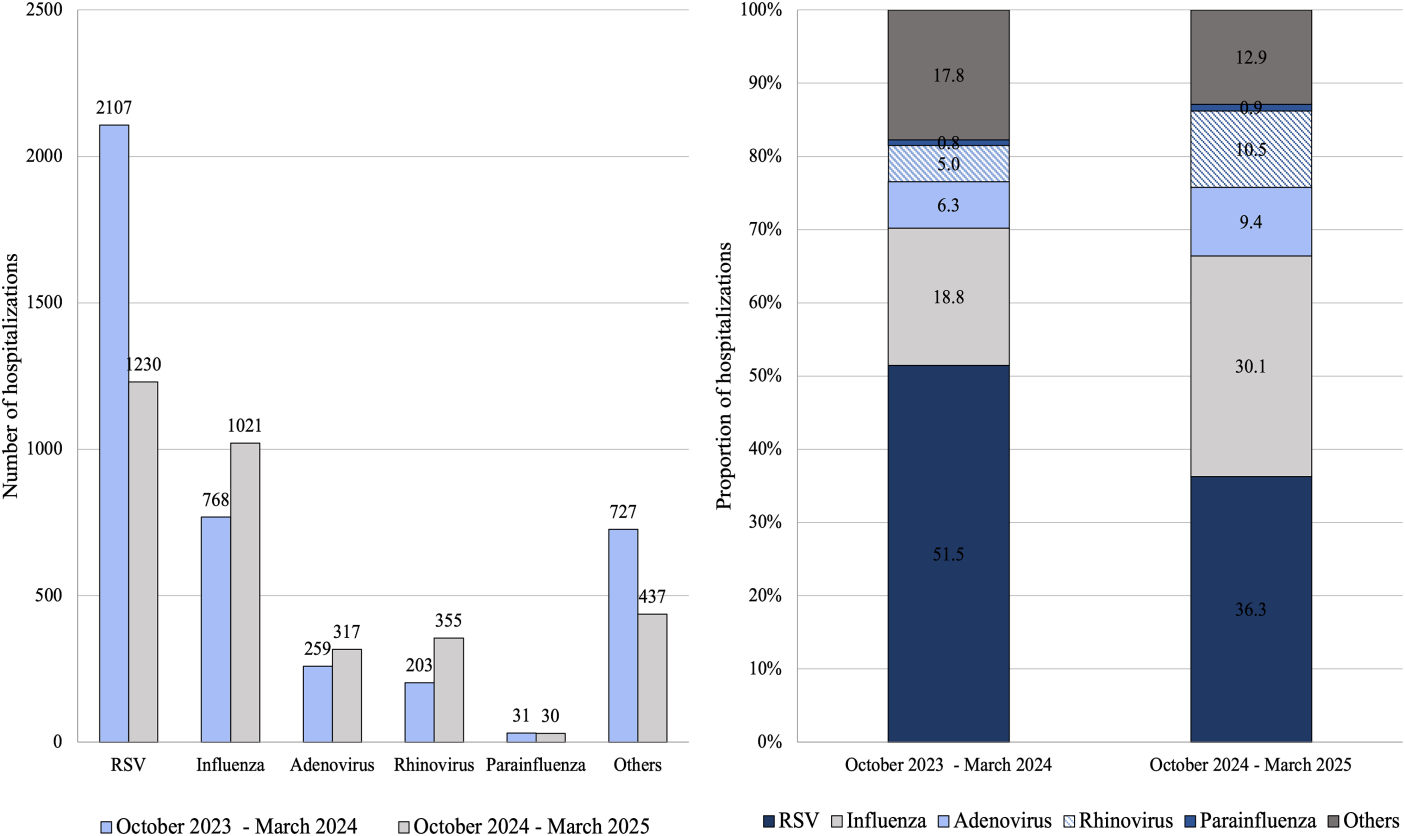
Hospitalizations (left panel) and proportion of hospitalizations (right panel) by type of virus in the two most recent autumn–winter seasons (October 2023 to March 2024; October 2024 to March 2025), the latter corresponding to the first with nirsevimab implementation. RSV: respiratory syncytial virus.

### 3.4 Hospitalization Trends

Hospitalization rates per 100,000 children under 18 years rose from 58.2 in 2022 to 87.4 in 2024, with the most pronounced increase occurring between 2022 and 2023 (+30.8%). RSV remained the main contributor, increasing from 22.2 to 35.5 per 100,000 (+59.9% overall). Other unspecified viral bronchiolitis also contributed substantially, rising from 12.3 to 18.6 per 100,000 (+51.0%). Influenza declined slightly in 2023 but increased again in 2024 (+31.7%, from 11.1 to 13.8 per 100,000). Adenovirus peaked in 2023, with a more than twofold increase (+109.8%, from 6.4 to 13.5 per 100,000), before decreasing in 2024, although it remained above the 2022 values. Rhinovirus remained stable through 2023 but nearly doubled in 2024 (+81.8%, from 5.1 to 9.3 per 100,000). Parainfluenza showed an increase in 2024 (+34.8%), but its contribution remained minimal (1.2 per 100,000) (Table 1).

## 4 Discussion

This multicenter study analyzed pediatric hospital admissions for ALRTIs between January 2019 and March 2025 in Italy, focusing on epidemiological changes after the COVID‐19 pandemic, which was officially declared over on May 5, 2023 [16], and during the first season of nirsevimab immunization. To our knowledge, this is the first multicenter national analysis to describe pediatric respiratory virus dynamics in Italy across the postpandemic period and the initial nirsevimab season, providing one of the earliest real‐world updates on these trends in Europe. While postpandemic epidemiological trends have been well‐documented in several studies, to our knowledge, this is among the first to update this evidence following the initial season of nirsevimab introduction. The profound effect of the COVID‐19 pandemic on respiratory virus epidemiology has been widely reported, with marked reductions in acute respiratory illnesses, community‐acquired viral pneumonia, and bronchiolitis coinciding with the global implementation of NPIs [2, 9, 17, 18]. These measures led to sharp declines in RSV and influenza activity, with the typical winter peaks disappearing during the 2020–21 season [9, 17, 18, 19, 20, 21, 22, 23]. Following the relaxation of NPIs, viral circulation resumed but displayed atypical dynamics, including shifts in the seasonal peaks [17, 18, 19, 20, 21, 22, 23]. Our findings are consistent with these reports, showing a marked decline in ALRTIs admissions in 2020–2021, followed by a progressive rise from autumn to winter 2021–2022, although with pathogen‐specific differences. RSV demonstrated a substantial drop after the January–February 2020 peak, with an early resurgence in November–December 2021 and again in December 2022. Peaks then shifted to later winter months, with a progressive decline culminating in a marked reduction during the 2024–2025 season. Similar patterns have been described in German and Norwegian studies [21, 24]. Conversely, influenza showed a delayed re‐emergence compared with persistently low levels until 2022, followed by a December peak in 2022, a January peak in 2024, and broader activity extending into March 2025. These findings align with reports from Germany and South Korea, suggesting that influenza circulation may be regaining pre‐pandemic seasonal patterns [21, 25]. Rhinovirus and adenovirus appeared less affected by NPIs, with only a transient decline in 2020, followed by rapid recirculation and steadily increasing activity. Rhinovirus was the first virus to re‐emerge after the onset of the pandemic maintaining its bimodal distribution with a dominant October surge [2, 9, 18, 19, 26, 27]. Adenovirus hospitalizations also rose steadily, surpassing prepandemic levels from 2022 onwards, consistent with trends observed in Japan and South Korea [28, 29, 30]. Changes in diagnostic practices should also be considered when interpreting these trends. The increasing availability and use of multiplex PCR panels following the COVID‐19 pandemic may have improved the detection of respiratory pathogens such as adenovirus and rhinovirus, potentially contributing to part of the observed increase in hospitalizations. Both viruses likely persisted due to intrinsic properties such as the absence of an envelope, high environmental stability, prolonged shedding, and extensive genomic diversity [9, 18, 19]. Parainfluenza virus contributed the smallest proportion of cases, showing a clear decline after 2019 and only minor, irregular seasonal activity thereafter, with occasional biannual patterns emerging from 2022 onwards. These findings are consistent with previous reports describing subtype‐specific variation and altered seasonal dynamics during and after the pandemic [9, 19, 31, 32].

The 2024–2025 autumn–winter season differed substantially from those of previous years. The defining feature was the introduction of RSV immunoprophylaxis with nirsevimab, in Italy as in other countries. Nirsevimab has shown consistent effectiveness in reducing RSV‐related hospitalizations in infants younger than 12 months in real‐world studies [10, 11, 12, 13, 14]. In our study, the overall decline in admissions was largely driven by a marked fall in RSV‐related hospitalizations, with monthly numbers consistently lower and peak activity nearly halved compared with 2023–24. By contrast, hospitalizations due to influenza, rhinovirus, and adenovirus increased. A prospective Spanish study reported an ≈ 45%–50% reduction in RSV‐related hospitalizations and a concurrent increase in influenza and rhinovirus detections following the introduction of nirsevimab [15]. Consistently, we observed a 41.6% reduction in RSV admissions during the 2024–2025 season compared with 2023–24, accompanied by increases in influenza, adenovirus, and rhinovirus. These parallel findings are consistent with possible postimmunoprophylaxis shifts in respiratory virus circulation, although causal relationships cannot be established from ecological observational data. However, alternative explanations should also be considered. For instance, changes in healthcare‐seeking behavior, hospital admission thresholds, or bed availability after the RSV peak might have influenced the relative increase of other viruses. Furthermore, the asynchronous and heterogeneous rollout of nirsevimab across Italian regions during the 2024–2025 season [33, 34] may have attenuated or obscured the overall magnitude of RSV reduction observed in this multicenter dataset, limiting the generalizability of these findings to settings with more uniform immunoprophylaxis coverage. In addition, substantial year‐to‐year variability is characteristic of respiratory virus circulation and may occur independently of immunization strategies. This is particularly relevant for less frequently detected pathogens such as parainfluenza virus, where relatively small absolute changes may generate large percentage differences. The apparent shift in pathogen proportions could therefore reflect both ecological and healthcare system dynamics. Nonetheless, the coherent temporal patterns observed across pathogens are consistent with mechanisms of viral interference, such as competition for susceptible hosts or transient cross‐protection mediated by interferon and other innate immune pathways [9, 15, 18, 19]. Although causal inference is beyond the scope of this retrospective design, these observations warrant further virologic and modeling studies to disentangle ecological from structural determinants [15]. From a public health perspective, these findings underscore the importance of maintaining and strengthening national respiratory virus surveillance systems that can effectively integrate laboratory, clinical, and epidemiological data. The marked decline in RSV hospitalizations following the introduction of nirsevimab, together with the increased circulation of other respiratory viruses, underscores the importance of continuous monitoring to assess potential shifts in pathogen dominance and timing of seasonal epidemics.

Such evidence is essential to optimize preparedness, inform vaccination and immunoprophylaxis strategies, and anticipate pediatric healthcare demand. Future studies integrating real‐time laboratory surveillance with age‐specific and severity‐stratified analyses will be crucial to assess the long‐term population‐level impact of nirsevimab and to elucidate its potential indirect effects on the ecology of non‐RSV respiratory viruses.

This study has several limitations. First, data collection began in 2019, preventing comparisons with longer pre‐pandemic periods. Second, the use of ICD‐9‐CM codes did not allow assessment of coinfections, intensive care admissions, or infection severity. Third, regional variability in the number of hospitalizations for RSV bronchiolitis may have arisen from the asynchronous rollout of nirsevimab immunoprophylaxis [33, 34]. In addition, stratified analyses by sex, age group, comorbidities, or infection severity were not feasible due to the absence of detailed clinical data. Age‐stratified analyses, particularly among infants younger than 12 months, were not available and therefore the direct impact of nirsevimab on its target population could not be assessed. Coding practices may also have varied across centers, introducing potential misclassification. COVID‐19‐specific ICD‐9‐CM codes were not included in the analysis, as they were introduced partway through the study period with heterogeneous adoption across centers, which would have introduced inconsistency in trend analyses. However, prior to the availability of dedicated codes, particularly during the first pandemic wave in early 2020, SARS‐CoV‐2‐associated respiratory infections may have been captured under nonspecific diagnostic categories, including the “Other” category. Furthermore, information on the number of PCR tests performed on pharyngeal swabs was not available, and testing practices likely differed over time and between hospitals, particularly following the adoption of multiplex assays during the pandemic [9, 18, 35]. Changes in diagnostic practices should also be considered when interpreting trends in adenovirus and rhinovirus detection, as observed increases may partly reflect improved detection rather than true changes in disease incidence. Moreover, this study was limited to hospital‐managed cases and did not capture community‐based infections or children managed exclusively in primary care. Natural year‐to‐year fluctuations in viral circulation may also occur independently of prophylactic or therapeutic interventions; therefore, longer‐term studies including multiple postnirsevimab seasons and incorporating outpatient and primary care data will be essential to confirm and contextualize these findings.

In summary, this multicenter national study depicts a rapidly evolving postpandemic landscape of pediatric respiratory virus circulation in Italy, marked by a substantial decline in RSV hospitalizations during the first nirsevimab season and concurrent increases in other viral pathogens. Collectively, these findings highlight the dynamic nature of viral ecology in children and underscore the importance of continuous, integrated surveillance to inform adaptive immunization policies and pediatric healthcare preparedness.

## Author Contributions

**Federica Attaianese:** conceptualization, investigation, funding acquisition, writing – original draft, methodology, validation, visualization, writing – review and editing, formal analysis, data curation, supervision, resources. **Silvia Campani:** conceptualization, investigation, funding acquisition, writing – original draft, methodology, validation, visualization, writing – review and editing, formal analysis, data curation, supervision, resources. **Sandra Trapani:** investigation, writing – original draft, methodology, validation, visualization, writing – review and editing, supervision, conceptualization, funding acquisition, formal analysis. **Ilaria Alberti:** investigation, writing – original draft, writing – review and editing, data curation. **Maurizio Aricò:** investigation, writing – original draft, writing – review and editing, data curation. **Marina Attanasi:** investigation, writing – original draft, writing – review and editing, data curation. **Giulia Bertolucci:** investigation, writing – original draft, writing – review and editing, data curation. **Silvia Bressan:** investigation, writing – original draft, writing – review and editing, data curation. **Désirée Caselli:** investigation, writing – original draft, writing – review and editing, data curation. **Veronica Casotto:** investigation, writing – original draft, writing – review and editing, data curation. **Salvatore Cazzato:** investigation, writing – original draft, writing – review and editing, data curation. **Francesco Chiarelli:** investigation, writing – original draft, writing – review and editing, data curation. **Enrico Felici:** investigation, writing – original draft, writing – review and editing, data curation. **Anna Frusciante:** investigation, writing – original draft, writing – review and editing, data curation. **Maria Antonia Galeone:** investigation, writing – original draft, writing – review and editing, data curation. **Silvia Garazzino:** investigation, writing – original draft, writing – review and editing, data curation. **Antonietta Giannattasio:** investigation, writing – original draft, writing – review and editing, data curation. **Eloisa Gitto:** investigation, writing – original draft, writing – review and editing, data curation. **Antonio Guerriero:** investigation, writing – original draft, writing – review and editing, data curation. **Fiorentina Guida:** writing – original draft, investigation, writing – review and editing, data curation. **Giovanna Iudica:** investigation, writing – original draft, writing – review and editing, data curation. **Marcello Lanari:** investigation, writing – original draft, writing – review and editing, data curation. **Claudia Mandato:** investigation, writing – original draft, writing – review and editing, data curation. **Alice Manzini:** investigation, writing – original draft, writing – review and editing, data curation. **Gaia Martelli:** investigation, writing – original draft, writing – review and editing, data curation. **Gregorio Paolo Milani:** investigation, writing – original draft, writing – review and editing, data curation. **Maria Moriondo:** investigation, writing – original draft, writing – review and editing, data curation. **Lucia Dora Notarangelo:** investigation, writing – original draft, writing – review and editing, data curation. **Felice Nunziata:** investigation, writing – original draft, writing – review and editing, data curation. **Roberta Pellegrino:** investigation, writing – original draft, writing – review and editing, data curation. **Diego Peroni:** investigation, writing – original draft, writing – review and editing, data curation. **Emanuela Piccotti:** investigation, writing – original draft, writing – review and editing, data curation. **Eduardo Ponticiello:** investigation, writing – original draft, writing – review and editing, data curation. **Silvia Ricci:** investigation, writing – original draft, writing – review and editing, data curation. **Immacolata Rulli:** investigation, writing – original draft, writing – review and editing, data curation. **Fabio Savoia:** investigation, writing – original draft, writing – review and editing, data curation. **Erika Silvestro:** investigation, writing – original draft, writing – review and editing, data curation. **Antonella Sisto:** investigation, writing – original draft, writing – review and editing, data curation. **Federica Soro:** investigation, writing – original draft, writing – review and editing, data curation. **Stefania Tonetto:** investigation, writing – original draft, writing – review and editing, data curation. **Alessandro Zago:** investigation, writing – original draft, writing – review and editing, data curation. **Carlo Dani:** investigation, writing – original draft, writing – review and editing, data curation. **Chiara Azzari:** investigation, conceptualization, writing – original draft, methodology, validation, writing – review and editing, visualization, supervision, data curation, formal analysis. **Giuseppe Indolfi:** conceptualization, investigation, funding acquisition, writing – original draft, methodology, validation, visualization, writing – review and editing, data curation, supervision, formal analysis, resources.

## Ethics Statement

This study used anonymized, aggregated hospital activity data and did not involve the collection or processing of identifiable patient information. In accordance with national and institutional regulations, it was considered exempt from full ethical review. No informed consent was required.

## Conflicts of Interest

Dr. Milani reported receiving grants from Angelini Pharma S.P.A, personal fees from Angelini Pharma S.P.A, and grants from Reckitt Benckiser Healthcare SPA outside the submitted work. Dr. Azzari reported receiving personal fees from Sanofi, Pfizer, and Moderna and grants from GSK and Merck outside the submitted work. No other disclosures were reported.

## Data Availability

De‐identified data and statistical analysis code will be made available upon reasonable request to the corresponding author for academic purposes, following approval of a written proposal and data sharing agreement.
